# The central region of the msp gene of Treponema denticola has sequence heterogeneity among clinical samples, obtained from patients with periodontitis

**DOI:** 10.1186/1471-2334-10-345

**Published:** 2010-12-07

**Authors:** Paolo Gaibani, Maria Teresa Pellegrino, Giada Rossini, Gualtiero Alvisi, Luisa Miragliotta, Carlo Prati, Vittorio Sambri

**Affiliations:** 1 Department of Hematology and Oncology "L. and A. Seragnoli", University St. Orsola-Malpighi Hospital, V. Massarenti 9, Bologna, Italy; 2 Department of Odontostomatological Sciences, University of Bologna, V. San Vitale 59, Bologna, Italy

## Abstract

**Background:**

*Treponema denticola *is an oral spirochete involved in the pathogenesis and progression of periodontal disease. Of its virulence factors, the major surface protein (MSP) plays a role in the interaction between the treponeme and host. To understand the possible evolution of this protein, we analyzed the sequence of the *msp *gene in 17 *T. denticola *positive clinical samples.

**Methods:**

Nucleotide and amino acid sequence of MSP have been determined by PCR amplification and sequencing in seventeen *T. denticola *clinical specimens to evaluate the genetic variability and the philogenetic relationship of the *T. denticola msp *gene among the different amplified sequence of positive samples. In silico antigenic analysis was performed on each MSP sequences to determined possible antigenic variation.

**Results:**

The *msp *sequences showed two highly conserved 5' and 3' ends and a central region that varies substantially. Phylogenetic analysis categorized the 17 specimens into 2 principal groups, suggesting a low rate of evolutionary variability and an elevated degree of conservation of *msp *in clinically derived genetic material. Analysis of the predicted antigenic variability between isolates, demonstrated that the major differences lay between amino acids 200 and 300.

**Conclusion:**

These findings showed for the first time, the nucleotide and amino acids variation of the *msp *gene in infecting *T. denticola*, *in vivo*. This data suggested that the antigenic variability found in to the MSP molecule, may be an important factor involved in immune evasion by *T. denticola*.

## Background

Periodontitis is a chronic inflammatory condition that is characterized by the progressive destruction of periodontal tissue [1]. This common infection is caused by polymicrobial flora, comprising several anaerobic, gram-negative bacteria. The oral spirochete *Treponema denticola *is often isolated from the affected sites and plays an important role in the polymicrobial pathogenesis of acute and chronic periodontal disease [2,3]. The outer membrane of *T. denticola *bears several antigens that control the interaction with host cells and tissues thus contributing to the pathogenesis of periodontal disease. In particular, the major surface protein (MSP) has been recently reported to alter the normal homeostasis of endothelial cells in vitro [4]. Further, MSP mediates the adhesion to and cytopathic effects of *T. denticola *on host cells [5,6]. MSP is a porin-like protein that has pore-forming activity, similar to other porins in the outer membrane of Gram-negative bacteria. MSP exists in an oligomeric form in the cell membrane of *T. denticola *and is homologous to the *T. pallidum *subsp. *pallidum *repeat (Tpr) proteins, which is a target of the antibody response during syphilis [6,7]. Recently, we demonstrated that specific polyclonal antibodies against MSP have strong opsonizing effects on the phagocytosis of *T. denticola *by murine macrophages in vitro [8]. Several studies have shown the ability of MSP to mediate the attachment to extracellular matrix (ECM) components, to induce the release of proteinase from human polymorphonuclear leukocytes [9,10], and to up regulate pro-inflammatory cytokines in different cells in vitro [11,12]. MSP complexes with chymotrypsin-like protein (CTLP) in the outer membrane of living *T. denticola *to form an oligomeric complex that has an apparent molecular mass of approximately 150 kDa [13]. The apparent molecular mass of isolated MSP ranges from 53 to 64 kDa, depending on the strain of *T. denticola *[5]. MSP is 543 residues long in ATCC 35405 strain and composed by 547 amino acids in the ATCC 33520 strain, which share an identical sequence homology [14] between the 3' and 5' ends and a low degree of homology in the central region. In the OTK strain of *T. denticola*, the amino acid sequences of MSP differ significantly with that of ATCC35405 and ATCC33520 strains [14]. Recently, Edwards and colleagues demonstrated that the central region of MSP mediates its binding to host extracellular matrix (ECM) components and that this area is the preferential target of host immune responses. These findings are consistent with the proposed cellular localization of the MSP antigen, wherein the central region is the only area of the molecule that is exposed on the surface of living *T. denticola *cells [10,15]. Because the central region of MSP mediates the effects of the entire protein during its interaction with the host, it is likely that differences in the amino acid sequence of this region affect its function differentially during infection. Aim of this study was to investigated for the first time, the nucleotide and amino acids sequence of the *msp *gene among infected *T. denticola *obtained from periodontal patients. As hypothesized for the Tprk antigen of *T. pallidum *that undergoes nucleotide variation following serial passages of *T. pallidum *in rabbit [16], we analyzed the nucleotide and amino acids variation of the *msp *gene in, *in vivo *infecting *T. denticola*. Based on these considerations, we analyzed the sequence of the *msp *gene in 17 clinical samples from acute periodontitis patients who were positive for *T. denticola *by real-time PCR [8].

## Methods

### Clinical samples selection

Seventeen subjects with chronic periodontitis were selected from a pool of patients who sought dental treatment at the Department of Oral and Dental Sciences, University of Bologna, Bologna. In addition, ten periodontally healthy volunteers participated in the study as controls. Periodontitis patients showed at least two sites with probing pocket depth and clinical attachment level of ≥4 mm and gingival recession. Healthy controls showed no sites with a probing pocket depth of >3 mm and clinical attachment level of <4 mm. Exclusion criteria included pregnancy, systemic conditions that could affect the progression or treatment of periodontal diseases, and use of antibiotics 6 months prior to entry into the study. All patients were informed about the nature of the study, and a signed consent form was obtained from each individual. The study was conducted following the ethical rules of University of Bologna. The sampling procedure has been reported [17].

The presence of *T. denticola *in these clinical specimens was determined by real-time PCR of 16S rRNA with the primers Dent1 and Dent2, as shown in Table 1 as reported previously [8], before the specific amplification of *msp*.

**Table 1:** Specific T. denticol a primers sets and cycling condition used in this study.

Primer	Nucleotide Sequence	Amplicon Size	Cycling conditions
Dent 1 Dent 2	5'TAATACCGAATGTGCTCATTTACAT3' 5'TCAAAGAAGCATTCCCTCTTCTTCTTA3'	316 bp	36 cycles 95°C 30" 60°C 1' 72°C 1'
			
Kx14 Kx04	5'GCTTGACAAGTGGATTTGGCTGTG3' 5'GAGAATAGCAGCAGAGTCTATTAG3'	1777 bp	30 cycles 94°C 1' 55°C 1' 72°C 3'
			
Kx14 Kx09	5'GCTTGACAAGTGGATTTGGCTGTG3' 5'CGAACGTCACCTTCGGTCTTTGAG3'	294 bp	31 cycles 94°C 1' 55°C 1' 72°C 1'
			
Td03 Td06	5'CTCAAAGACCGAAGGTGACGTTCG3' 5'GCATATTTGTTTGCTGCG3'	571 bp	31 cycles 94°C 1' 55°C 1' 72°C 1'
			
Td05 Kx04	5'CCGCAGCAAACAAATATGC3' 5' GAGAATAGCAGCAGAGTCTATTAG3'	875 bp	31 cycles 94°C 1' 55°C 1' 72°C 2'

### Nucleic acid extraction

Bacterial DNA was purified from paper conical tip swabs with the automated NucliSENS EasyMAG (Biomerieux, France) extractor according to the manufacturer's instructions.

### PCR amplification and sequencing

The amplification of *msp *was performed using the Kx14 and Kx04 primers, located in the 5' and 3' end flanking regions of the *msp *open reading frame (ORF), using reported conditions [14]. Individual nested PCR reactions were used to amplify regions of *msp *separately (5'end, 3' end, and the central region), using the Kx14 primer with Kx09 for the 5'end, the primer set Td03 and Td06 for the central fragment, and the Td05 and Td04 primers for the 3'end. For a complete and detailed description of the primer sets, see Table 1. The amplification mixture (50 μl final volume) contained 2 μM dNTPs, 0.2 mM of each primer, 3 mM MgCl_2 _, 1 UI of Taq DNA polymerase (Fermentas Life Science, Canada), and 5 μl of template DNA. Cycling conditions are reported in Table 1. The amplicons were purified using the PureLink Quick Gel Extraction Kit (Invitrogen, Italy) and sequenced (PRIMM, Italy).

### Phylogenetic and predicitive antigenicity variability analysis

Multiple alignment was generated with ClustalW software (version 2, available at: http://www.ebi.ac.uk/clustalw2), and a phylogenetic tree was constructed with bootstrap resempling analysis (1000 interactions) by using neighbor-joining algoritm implemented in MEGA 4 software [18]. All 17 *msp *gene sequences were deposited in GenBank with the following accession numbers: B5: GU946067; B6: GU946068; B14: GU946069; B16: GU946070; B20: GU946071; B21: GU946072; B22: GU946073; B23: GU946074; B30: GU946075; B34: GU946076; B37: GU946077; B40: GU946078; B46: GU946079; B52: GU946080; B66: GU946081; B69: GU946082; B74: GU946083. The putative antigenic variability in the 17 *msp *sequences was evaluated in silico by analyzing entire individual amino acid MSP sequences with CLC Bio Workbench [19].

## Results

### Nucleotides and amino acids variability of the Major Surface Protein coding sequence

**Figure 1:**
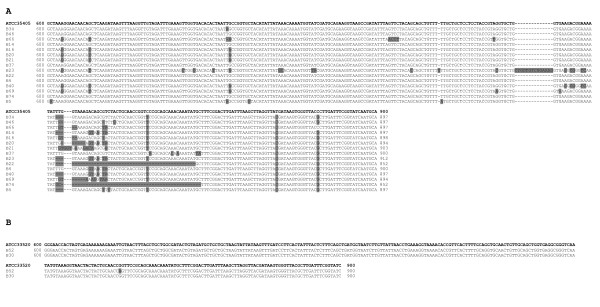
**Diversity of *msp *sequences**. Sequence alignments of 17 central regions (from 600 to 900 nucleotides) from *T. denticola *positive clinical specimens. In panel A are shown the clinical samples of Group A. In panel B are shown the clinical samples of Group B. The upper line contains the sequence of *T. denticola *strain ATCC 35405 and ATCC 33520, respectively, in both panels. The grey areas indicate variations of single nucleotide positions compared with *T. denticola *ATCC 35405.

The entire *msp *gene (ORF) derived from 17 *T. denticola *positive samples, obtained from Periodontitis patients, was amplified by PCR and sequenced in order to determine the extent of sequence variability. Controls patients resulted negative for the presence of *T. denticola *16S rRNA analyzed by specific PCR, as previously described [8]. The sequences of *msp *from 15 samples (defined as Group A) showed high homology (≥98%) to that of ATCC *T. denticola *strain 35405 (GenBank accession number U29399), and the remaining 2 isolates (group B) matched more closely with ATCC strain 33520 (Gen Bank accession number U66255). No sample shared homology with the *T. denticola *strain OTK (GenBank accession number U66256). Notably, sequence analysis of the entire *msp *gene in all 17 samples showed that the 5'region, encompassing base pairs 1-600, was 99% homologous to that of *T. denticola *ATCC 35405 and ATCC 33520, as was observed for the 3' region, comprising nucleotides 900-1632. The highest variability existed in the central region of *msp *gene between nucleotides 600 and 900 in group A samples (Figure 1). No such differences were seen in Group B specimens.

**Figure 2:**
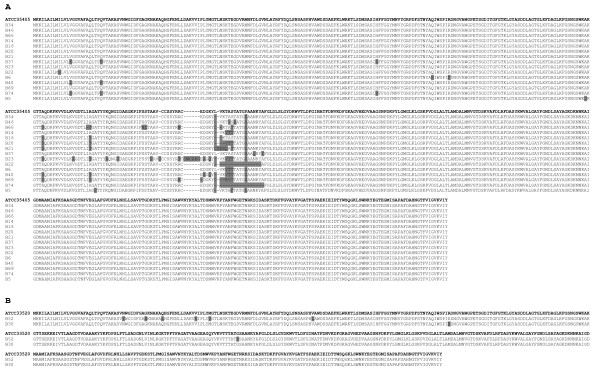
**MSP amino acid sequence alignment of *T. denticola *strains ATCC 35405 and Group A (panel A), and ATCC 33520 and Group B (panel B)**. The grey areas indicate single amino acid substitutions compared with ATCC 35405 (panel A) and ATCC 33520 (panel B).

The influence of silent and nonsilent nucleotide mutations on the variability of putative amino acid sequences was evaluated by comparing the polypeptides of MSP sequences from *T. denticola *strains ATCC 35405 and ATCC 33520 (Figure 2). The level of amino acid homology ranged between 92% and 99%. In Group A, the majority of mismatches were distributed along the entire length occurred between amino acids 200 and 300. In Group B, the homology was greater than 98% (Figure 2). These results confirmed for the clinical samples, the findings reported by Fenno and co-workers, and obtained with the two laboratory adapted strains of *T. denticola *ATCC35405 and ATCC33520 [14], that the highest amino acidic variability lays in the central region of MSP. Moreover, interestingly, the unprocessed peptide of 6 isolates, (B34, B66, B37, B23, B22, B69 and B74) showed the highest difference in the aminoacidic identity, with an homology comprised between 92,1% and 97,2% to the ATCC 35405 MSP (more than 50% of the total amino acidic substitutions resulted not conservative). In opposite, the remaining 11 clinical isolates showed an homology over 98% (the mean percentage of conservative or neutral amino acidic substitutions was 75%) to ATCC35405 and ATCC33520, as showed in Figure 2.

### Phylogenetic analysis

**Figure 3:**
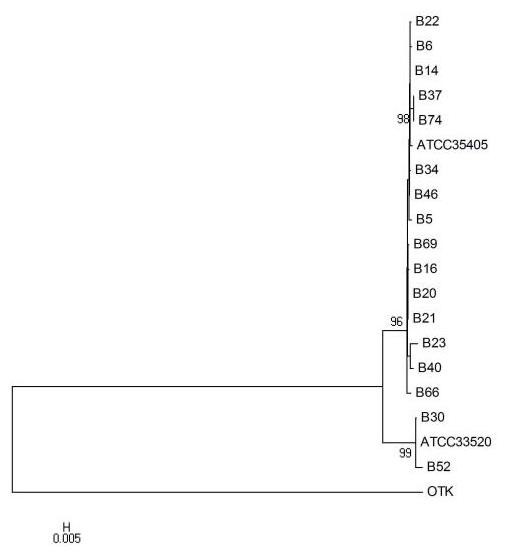
**Evolutionary relationships of *T. denticola msp *gene, deduced from the sequences of 17 clinical samples, ATCC 35405, ATCC 33520, and OTK**. The phylogenetic analysis was performed using the neighbor-joining method with MEGA 4 on aligned sequences from the *msp *complete cds sequence (bootstrap values >75 are shown at nodes).

Phylogenetic analysis performed on nucleotide sequences revealed that the identified variants are not randomly distributed, but can be clearly clustered into two distinct group, as shown in Figure 3. Based on these findings, the 17 samples were divided into 2 distinct groups, A (15 samples) and B (the remaining 2), whose *msp *nucleotide sequences were closely related to *T. denticola *ATCC 35405 and ATCC 33520, respectively. The phylogenetic analysis of *msp *sequences was also extended to the OTK strain, demonstrating that this last isolate diverged from Groups A and B. In addition, phylogenetic trees of the rooted *msp *sequences demonstrated a low evolutionary rate among the different samples (Figure 3).

### Antigenicity analysis

**Figure 4:**
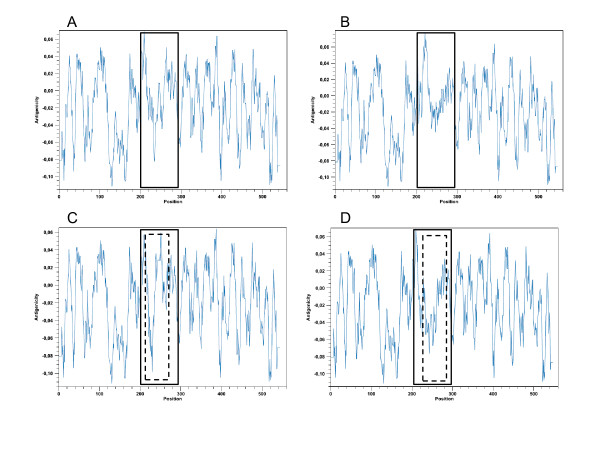
**Antigenicity plot of *T. denticola *strains ATCC 35405 (frame A), ATCC 33520 (frame B), and 2 representative specimens (B66, frame C; B23, frame D)**. In each frame, the area of the plot that is surrounded by a continuous line represents the central region of MSP. The internal smaller area, surrounded by the dotted line, represents the portion that has the greatest difference in predicted antigenicity between specimens B66 and B23 and ATCC35405.

To determine whether any putative amino acid substitutions determined an antigenic variation on each MSP sequences derived from clinical samples, an in silico analysis was performed by using CLC Main Workbench 4.1.1 Software program. The predicted antigenicity of the entire MSP molecule did not differ between 10 of 17 clinical specimens and that of ATCC 35405 and ATCC 33520. The remaining 7 clinical samples (all from Group A: B74, B40, B21, B22, B23, B66, and B69) showed predicted, yet variable, antigenic variation between amino acid positions 200-300. Figure 4 shows the predicted antigenicity plots of ATCC 35405, ATCC 33520, and the 2 most disparate clinical samples (B66 and B23). In these clinical isolates, the antigenic peak shifted in a diverse positions (Figure 4 marked with dotted line) produced by substitution of an amino acid with diverse functionality (polar to non-polar or vice versa). In addition, the antigenicity plot clearly showed that the major difference were present among the central region of the deduced MSP molecule.

## Discussion

Outer membrane-exposed antigens regulate many interactive processes between *T. denticola *and host cells and tissues. In this study, we demonstrated that the central region of the *msp *gene experiences the highest degree of variability compared with the 5'and 3' ends in PCR-positive clinical samples from patients who suffer from acute periodontal disease. These data confirm the high conservation in the terminal ends of *msp *that has been reported for *T. denticola *strains that have been cultured under laboratory conditions for long periods [14].

The central area of the MSP polypeptide has recently been demonstrated to be exposed to the surface of living *T. denticola *cells and act as the primary target for immune responses. In particular, Edwards and colleagues reported that this region contains epitopes that bind to immune serum against recombinant MSP from the homologous strain ATCC 35405 [10]. We recently reported that specific immune responses against whole native MSP affects the efficacy of *T. denticola *phagocytosis by isolated murine macrophages under anaerobic conditions in vitro [8].

The variability in MSP amino acid sequences in the clinical samples in this study could influence the evolution of periodontitis, because the efficacy of a patient's immune response during in vivo infections might be hampered. The interaction between *T. denticola *and the host immune response could influence sequence variations into the surface-exposed portion of MSP, corresponding to the central region of *msp *gene. This immune-driven sequence variability in one of the most prominent virulence factors of *T. denticola *might underlie the pathogenic mechanisms that govern the development of periodontal diseases in vivo. Further, *T. denticola *migrates from the oral cavity to other anatomical sites [20]. This property of wild-type infectious treponemes could be linked to variations in the surface-exposed portion of MSP. In addition, amino acid variations that were detected in 7 clinical specimens led to antigenic modification of the surface-exposed portion of MSP. The antigenic variation in surface-exposed polypeptides is a common pathogenic feature of spirochetes. In particular, the outer membrane lipoprotein P66 of *Borrelia *spp. has a surface-exposed loop that is subject to selective pressure by antibodies, resulting in sequence and size variations [21]. The antigenic differences in the most prominent surface-exposed protein of 2 strains of the avian pathogenic *Borrelia anserina *impede strain-specific antibody-mediated protection against infections [22]. MSP shares high homology with the Tprk antigen of *T. pallidum *subsp. *pallidum *[23]. The TprK protein is a target of opsonizing antibodies and protective immunity and is subject to immunologically driven sequence variation [7]. Centurion-Lara and colleagues demonstrated that the *tprK *gene undergoes nucleotide variation following serial passages of *T. pallidum *in rabbit [16]. *tprK *gene is highly variable within *T. pallidum *strains, and the evidence that its V regions elicit variant-specific antibody responses supports the hypothesis that TprK variants help organisms avoid immune responses in infected individuals, contributing to the ability of *T. pallidum *to effect chronic infection [24].

## Conclusion

Before our study, no sequence or related antigenic variations had been reported in *T. denticola msp *between patients. The differences in amino acid sequences of MSP that were predicted in this study can consistently modify its function during the pathogenesis of periodontal disease with regard to binding capacity to cells and extracellular matrix and to evasion of host responses by *T. denticola*. Further studies are necessary to demonstrate that the predicted antigenic variability of MSP regulates the pathogenesis of chronic periodontal disease, as has been hypothesized for the Tprk antigen of *T. pallidum *during the development of syphilis.

## Competing interests

The authors declare that they have no competing interests.

## Authors' contributions

All authors read and approved the final manuscript. PG, overviewed the work, performed the sequencing analysis and wrote the manuscript. MTP and LM conducted the PCR amplification. GR and GA discussed the data and helped in reviewing the manuscript. CP supervised the study. VS planned the experiments, supervised the study and wrote the paper

## Pre-publication history

The pre-publication history for this paper can be accessed here:

http://www.biomedcentral.com/1471-2334/10/345/prepub
